# Ultrasound assessment of rectus femoris and anterior tibialis muscles in young trauma patients

**DOI:** 10.1186/s13613-017-0326-x

**Published:** 2017-10-06

**Authors:** Maria Giuseppina Annetta, Mauro Pittiruti, Davide Silvestri, Domenico Luca Grieco, Alessio Maccaglia, Michele Fabio La Torre, Nicola Magarelli, Giovanna Mercurio, Anselmo Caricato, Massimo Antonelli

**Affiliations:** 1 Department of Anesthesia and Intensive Care, Fondazione Policlinico Universitario ‘A.Gemelli’, Largo A.Gemelli, 8, 00168 Rome, Italy; 2 Department of Surgery, Fondazione Policlinico Universitario ‘A.Gemelli’, Rome, Italy; 3 Department of Radiology, Fondazione Policlinico Universitario ‘A.Gemelli’, Rome, Italy

**Keywords:** Muscle mass, Muscle ultrasonography, Enteral feeding, Trauma

## Abstract

**Purpose:**

Quantitative and qualitative changes of skeletal muscle are typical and early findings in trauma patients, being possibly associated with functional impairment. Early assessment of muscle changes—as evaluated by muscle ultrasonography—could yield important information about patient’s outcome.

**Methods:**

In this prospective observational study, we used ultrasonography to evaluate the morphological changes of rectus femoris (RF) and anterior tibialis (AT) muscles in a group of young, previously healthy trauma patients on enteral feeding.

**Results:**

We studied 38 severely injured patients (median Injury Severity Score = 34; median age = 40 y.o.) over the course of the ICU stay up to 3 weeks after trauma. We found a progressive loss of muscle mass from day 0 to day 20, that was more relevant for the RF (45%) than for the AT (22%); this was accompanied by an increase in echogenicity (up to 2.5 by the Heckmatt Scale, where normal echogenicity = 1), which is an indicator of myofibers depletion.

**Conclusions:**

Ultrasound evaluation of skeletal muscles is inexpensive, noninvasive, simple and easily repeatable. By this method, we were able to quantify the morphological changes of skeletal muscle in trauma patients. Further studies may rely on this technicque to evaluate the impact of different therapeutic strategies on muscle wasting.

## Background

Muscle wasting is a frequent finding in critically ill patients and is associated with worse short- and long-term outcomes. Loss of mass and function of skeletal muscles starts early—in the first 24 h after admission to intensive care unit (ICU)—and may persist for years (‘post-ICU syndrome’). Loss of muscle mass is a major cause of ICU-acquired muscle weakness and is associated with delayed weaning, prolonged ICU and hospital stay and is an independent predictor of 1-year mortality [1–3]. Long-term muscle impairment may be responsible of physical, mental and cognitive dysfunction, which affects the quality of life of ICU survivors and increases the costs of the healthy care services [4–6]. Early physical rehabilitation has been associated with conflicting results in terms of functional outcome [7, 8], so that the best strategy would theoretically be to avoid or minimize muscle loss during ICU stay, for example delivering an appropriate nutritional support. Unfortunately, limited data clarify the possible impact of adequate calories and protein delivery on skeletal muscle preservation and long-term outcome of muscular function [9–11]; also, the conclusions of the few available clinical studies are controversial. Some studies have even suggested that increasing protein intake in the early phase of critical illness may accelerate muscle loss during the first week [4, 12].

The sequential assessment of quantitative and qualitative changes of muscle mass may help identify critically ill patients with high risk of muscle dysfunction, as well as verify the effects of different nutritional regimens. In this regard, B-mode ultrasonographic evaluation of skeletal muscles (in particular, rectus femoris and anterior tibialis) is an emerging and reliable tool to assess muscle changes over time. It is a bedside technique, easy to use and inexpensive [13]. Its cost-effectiveness is higher than CT scan evaluation, which has been used for the same purpose [2].

In this prospective clinical study, we evaluated the feasibility of detecting the quantitative and qualitative changes of rectus femoris and anterior tibialis muscles over the course of the ICU stay up to 20 days from admission in a cohort of young trauma patients.

## Methods

This was a prospective observational study performed in a cohort of severe multiple trauma patients admitted to the 20-bed ICU of our institution (Fondazione Policlinico ‘A.Gemelli’—University Hospital) in a period of 10 consecutive months. All subjects were on enteral feeding.

### Patients

We enrolled exclusively young trauma patients with an injury severity score(ISS) exceeding 25, who were admitted to our ICU within few hours after the injury. We recruited only well-nourished, previously healthy subjects, aged 18–59 y.o, with no past history of nutritional problems, chronic use of drugs nor orthopedic issues (such as skeletal fractures or immobilization) in the previous 2 years. Trauma patients not fulfilling this criteria were not considered for the enrollment. Exclusion criteria were: relevant comorbidities (renal, liver or heart disease or COPD), previous immune abnormalities (including treatment with corticosteroids), neuromuscular disease, past or recent history of cancer.

For each patient, demographics and clinical data were recorded: age, height, weight, body mass index (BMI), ISS, APACHE II score, Sequential Organ Failure Assessment score (SOFA), Glasgow Coma Scale (GCS)—both total and motor (GCS-M)—Glasgow Outcome Scale (GOS), number of days on mechanical ventilation, ICU length of stay, incidence of secondary infections, daily provision of calories and protein, blood levels of albumin, total protein and creatinine, blood urea nitrogen (BUN) and other laboratory data. Among mechanically ventilated patients, weaning was classified as simple, difficult or prolonged, as previously described [14]. Infections were classified according to definitions by the Center for Disease Control and Prevention [15].

### Nutritional support

Enteral feeding was started as soon as the patient was hemodynamically stable and fully resuscitated, usually within 24 h. Our nutritional target was to achieve a minimal protein intake of 0.8 g/kg/day within day 5 after admission. Patients who did not reach this target for any reason (gastrointestinal intolerance or contraindication to enteral feeding or repeated forced suspensions of enteral feeding because of multiple surgical procedures) were excluded from the final analysis. We used either a standard feeding formula (1.5 total kcal/ml, protein 60 g/L) or a high-protein formula (1.35 total kcal/ml, protein 75 g/L): These different regimens were not assigned by randomization, but they were the result of a change of nutritional policies in our ICU during the period of the study; though, the two groups of patients—nourished by a standard formula (SF) or nourished by a high-protein formula (HPF)—were similar in terms of age, Injury Severity Score (ISS), Acute Physiology and Chronic Health Evaluation (APACHE II score), height and weight. Tube feeding was started at a very low rate (10–20 ml/h) and increased at each 24-h interval as tolerated, in order to meet a minimal protein intake of 0.8 g/kg/day. The patient’s intolerance to feeding was defined on the basis of clinical signs (abdominal distention, vomiting, increase in serum lactate, high gastric residual volume).

### Ultrasonography of skeletal muscles

An ultrasound (US) evaluation of the rectus femoris (RF) and anterior tibialis (AT) muscles was performed in all patients at day 0 (within 24 h from trauma), 5, 10, 15 and 20. We used an US device with a 5- to 7.5-MHz linear probe (Esaote MyLab). According to a previously described methodology [13, 16], skeletal muscles were evaluated by US scan, collecting both quantitative and qualitative data. The transducer was placed perpendicular to the long axis of the muscle (i.e., perpendicular to the major axis of the limb), at 3/5 of the distance between the anterior superior iliac spine and the superior border of patella (i.e., about 15 cm from the patella) for the RF and 5 cm below the peroneal head for the AT muscle. The measurement points were marked with indelible ink to ensure day-to-day consistency and facilitate subsequent measurements. Visualization was consistently obtained, while the patients were supine with both legs in passive extension. Two measurements were taken for each muscle in each leg. In the presence of lower limb fractures, measurements were taken on the contralateral leg only. Excess contact gel was applied so to minimize underlying soft tissue distortion. One operator only (specifically trained in muscle ultrasonography) performed all measurements. US settings (depth, gain and focus) were standardized for both RF and AT examinations. After freezing the US image, quantitative parameters were recorded for both muscles: anterior–posterior diameter (AP diam); lateral–lateral diameter (LL diam); and cross-sectional area (CSA) (computed from the perimetral contour of the muscle section). The value of CSA is considered to be proportional to the total mass of the skeletal muscle [13, 16]. We also recorded one qualitative parameter—echogenicity—that was expressed according to the Heckmatt Scale [17]. Echogenicity of normal muscle is expected to be 1 on this scale. Increased echogenicity is usually regarded as an index of myofibers depletion [18].

*Primary endpoints* of our study were the qualitative and quantitative changes of skeletal muscles during 3 weeks of ICU stay, taking into consideration the role of protein intake. The study protocol was approved by the Ethics Committee of our institution (Prot. 10917/15).

### Statistical analysis

Qualitative data are expressed as number of events (%) and continuous data as median [interquartile range]. Difference in the distribution of qualitative variables in SF versus HPF group was investigated with the Chi-square test or Fisher’s exact test, as appropriate. Difference in the distribution of quantitative and ordinal variables in SF versus HPF group was assessed with the Mann–Whitney test. In the overall population, the significance of changes in the quantitative variables over time was determined with the one-way ANOVA test for repeated measures; paired comparisons between 2 consecutive timepoints were then analyzed with the Wilcoxon sum of ranks test. The effect of SF versus HPF on the change in the quantitative variables over time was determined with the two-way ANOVA. Paired comparisons between the distribution of quantitative variable in SF versus HPF group at each time point were also assessed with the Mann–Whitney test.

The entire analysis was conducted applying a bilateral null hypothesis; accordingly, results with two-tailed *p* ≤ 0.05 were considered significant.

Statistical analysis was conducted with SPSS 20.0.

## Results

A total of 120 trauma patients admitted to our ICU were screened for possible inclusion in our prospective study, and only 52 met the requirements. Nine patients did not reach the minimal protein intake by enteral feeding. Of the remaining patients, five died during the first 3 weeks. The final analysis was conducted on 38 patients, whose characteristics are listed in Table 1. All patients were young (median age 40 year old); most were male (76%), and all of them had a good nutritional status on admission (median BMI = 25). All patients were severely injured (ISS = 34, APACHE II score = 16), and most of them had associated brain injury (84%). There were no significant differences between patients on SF versus HPF.

**Table 1:** Demographics

	ALL	SF *n* 20	HPF *n* 18
Female sex, *n* (%)	9 (24)	5 (25)	4 (22)
Age (years)	40 [31–54]	47 [37–58]	37 [29–46]
Height (cm)	175 [167–180]	175 [168–180]	170 [167–180]
Weight (k)	75 [70–81]	80 [70–90]	75 [67–85]
BMI	25 [23–28]	26 [23–29]	23 [24–26]
GCS at inclusion	7 [3–9]	7 [3–9]	6 [3–10]
GCS-M at inclusion	4 [1–5]	4 [1–5]	4 [1–5]
Injury Severity Score (ISS)	34 [27–42]	34 [28–40]	34 [27–45]
Apache II score	16 [13–20]	16 [13–19]	18 [12–22]
SOFA	7 [5–9]	8 [5–10]	6 [5–9]
Brain injury *n* (%)	32 (84)	17 (85)	15 (83)
Thoracic trauma *n* (%)	30 (79)	16 (80)	14 (78)
Abdominal trauma *n* (%)	17 (45)	9 (45)	8 (44)
Pelvic trauma *n* (%)	15 (40)	8 (40)	7 (40)
Spinal trauma *n* (%)	21 (55)	10 (50)	11 (61)
GOS 28 days	3 [3, 4]	3 [3, 4]	3 [3, 4]
GCS-M at discharge	6 [4–6]	6 [5, 6]	6 [4–6]
Tracheostomy *n* (%)	24 (63)	12 (60)	12 (67)
Weaning			
Simple, *n* (%)	21 (55)	10 (50)	11 (61)
Difficult, *n* (%)	11 (29)	6 (30)	5 (28)
Prolonged, *n* (%)	6 (16)	4 (20)	2 (11)
Days of mechanical ventilation	13 [11–19]	14 [9–22]	13 [11–17]
Patients with at least one documented infection, *n* (%)	34 (90)	19 (95)	15 (83)
Patients with at least one documented infection MDR infection, *n* (%)	23 (61)	13 (65)	10 (56)
Patients with septic shock during the ICU stay, *n* (%)	7 (18)	5 (25)	2 (11)
ICU length of stay	22 (17–33)	22 (16–37)	22 (17–31)
ICU outcome, died, *n* (%)	3 (8)	2 (10)	1 (6)

Data expressed as median [interquartile range], if not otherwise specified

See abbreviations in the text

Muscular changes are shown in Table 2.

**Table 2:** Muscle ultrasound

	Day 0	Day 5	Day 10	Day 15	Day 20
RF: AP diam (mm)					
All pts	17 [15–20]	17 [15–20]	16 [13–19]	14 [12–17]	13 [9–15]
SF	17 [15–20]	18 [16–20]	19 [14–20]	15 [12–17]	14 [9–17]
HPF	17 [15–19]	16 [15–20]	16 [12–18]	13 [12–17]	9 [9–12]
RF: LL diam (mm)					
All pts	43 [40–46]	41 [38–44]	40 [37–45]	40 [36–44]	38 [34–41]
SF	41 [40–44]	41 [37–42]	39 [35–42]	40 [34–44]	35 [34–40]
HPF	43 [40–47]	44 [37–46]	44 [37–47]	40 [37–47]	41 [37–43]
RF: CSA (cm^2^)					
All pts	6.1 [5.1–7.3]	5.9 [4.8–6.3]	5.1 [4.3–6.2]	4.6 [3.8–5.3]	3.5 [3.2–4.7]
SF	6.1 [5.1–7.3]	5.9 [4.9–6.3]	5.6 [4.2–6.2]	4.7 [3.9–5.4]	3.5 [3.2–4.8]
HPF	6.3 [4.7–7.3]	5.9 [4.3–6.5]	5 [4.2–6.6]	4.4 [3.5–5.1]	3.5 [2.8–4.4]
RF: echogenicity, Heckmatt Scale					
All pts	1 [1, 2]	2 [1, 2]	2 [1–2.5]	2 [1–3]	2.5 [1.5–3]
SF	1 [1, 2]	2 [1, 2]	2 [1.3–2.8]	2.3 [1.6–3.5]	2.5 [2, 3]
HPF	1.3 [1, 2]	1.5 [1, 2]	1 [1, 2]	1.5 [1–2.5]	2.5 [1–3]
AT: AP diam (mm)					
All pts	22 [20–25]	21 [19–23]	20 [17–22]	19 [17–22]	18 [16–20]
SF	22 [20–24]	21 [19–24]	20 [18–23]	20 [17–23]	19 [15–21]
HPF	24 [20–26]	22 [19–23]	21 [17–22]	18 [17–20]	18 [18]
AT: LL diam (mm)					
All pts	25 [22–26]	24 [21–26]	23 [21–25]	22 [20–25]	22 [20–27]
SF	25 [23–27]	24 [21–27]	23 [21–26]	23 [19–26]	25 [21–28]
HPF	23 [22–26]	24 [21–26]	22 [21–25]	21 [20–24]	22 [20–22]
AT: CSA (cm^2^)					
All pts	5.6 [4.5–6.4]	4.8 [3.7–5.6]	4 [3.7–5.2]	4 [3.3–4.8]	4.2 [3.4–4.7]
SF	5.7 [4.7–6.8]	4 [3.6–6]	3.9 [3.7–5.2]	4 [3.3–5.7]	4 [3.2–5.2]
HPF	5.5 [4.5–6.2]	4.9 [4.2–5.9]	4.2 [3.6–5.3]	3.9 [3.3–4.5]	4.3 [3.4–4.6]
AT: echogenicity, Heckmatt Scale					
All pts	2 [1, 2]	2 [1–3]	2 [1–3]	1.5 [1–3]	2.5 [1–4]
SF	1.8 [1–2.1]	2.3 [1.3–3]	2 [1.3–3]	2 [1–3.4]	3.5 [1–4]
HPF	2 [1, 2]	2 [1–3]	1.3 [1–3]	1.3 [1–2.5]	1.5 [1–3]

Data expressed as median [interquartile range]

See abbreviations in the text

See statistical significance in the text

Results concerning muscular changes over the course of the study are reported in table 2. The RF muscle mass changed significantly during the ICU stay in all patients. Its AP diameter decreased progressively (ANOVA for repeated measures: *p* = 0.03), in particular from day 5 to day 20 (*p* < 0.05), though such decrease was not significant between day 0 and day 5 (*p* = 0.24). The LL diameter did not show a significant progressive decrease (ANOVA for repeated measures: *p* = 0.25), but the difference between day 0 and day 20 was significant (*p* = 0.04). The CSA of RF muscle progressively decreased during the ICU stay (ANOVA for repeated measures: *p* = 0.03), with a statistically significant difference among all time points between day 5 and day 20 (all *p* < 0.05), but not between day 0 and day 5 (*p* = 0.13). In particular, there was an overall 45% reduction in CSA during the first 20 days of ICU stay (15% loss from day 5 to 10, 12% from day 10 to 15, 21% from day 15 to 20).

As regards the AT muscle, its AP diameter decreased progressively during the ICU stay (ANOVA for repeated measures: *p* = 0.03) in all patients, with a statistically significant reduction among all time points between day 0 and day 20 (*p* < 0.05). Its LL diameter did not decrease significantly during the ICU stay (ANOVA for repeated measures: *p* = 0.63) but only between day 5 and 10 (*p* = 0.03). The 22% decrease in CSA of AT muscle during the overall ICU stay was not significant (ANOVA for repeated measures: *p* = 0.30).

There was a progressive increase in both RF and AT echogenicity—as evaluated with the Heckmatt Scale—from day 0 on (*p* < 0.05), with the main increase from day 0 to day 5.

None of these quantitative and qualitative muscular changes showed any significant difference between the groups SF versus HPF.

Nutritional intake is shown in Table 3. We had 20 patients in the SF and 18 in the HPF group. Mean protein intake after day 5 was of 0.87 g/kg/day in the SF group and 1.6 g/kg/day in the HPF group. Mean total calories were 19 kcal/kg in the SF group and 30 kcal/kg in the HPF group. No major differences were detected in the main laboratory values (Table 4), though HPF patients had nonsignificantly higher blood protein levels (*p* < 0.07) and significantly higher albumin levels (*p* < 0.03) at day 20.

**Table 3:** Nutritional intake

	Day 0–5	Day 5–10	Day 10–15	Day 15–20	SF versus HPF *p* value
Enteral nutrition (ml/day)					
SF	850 [625–900]	1000 [1000–1400]*	1050 [950–1500]*	1350 [1000–1500]*	0.007
HPF	1000 [700–1200]	1500 [1175–1500]*	1500 [1500–1700]*	1500 [1500–2000]*	
Proteins (g/day)					
SF	47 [32–63]*	67 [50–91]*	70 [48–90]*	70 [50–90]*	0.001
HPF	74 [40–93]*	112 [99–136]*	136 [112–136]*	120 [106–136]*	
Total calories (kcal/day)					
SF	1059 [763–1200]*	1500 [1160–1915]*	1500 [1050–2062]*	1500 [1250–2200]*	0.005
HPF	1326 [934–1564]*	1958 [1762–2250]*	2250 [1920–2341]*	2250 [1920–2560]*	

Data expressed as median [interquartile range]

The overall *p* value refers to the comparison between the whole time series of changes SF versus HPF

* *p* < 0.05 for the comparison of SF versus HPF at each time point

See abbreviations in the text

**Table 4:** Laboratory data

	Day 0	Day 5	Day 10	Day 15	Day 20	SF versus HPF p value
BUN (mg/dl)						
SF	12 [10–15]	17 [13–26]	20 [17–30]	25 [17–35]	24 [18–47]	0.51
HPF	13 [11–17]	18 [12–22]	23 [19–29]	20 [16–24]	30 [23–37]	
Creatinine (mg/dl)						
SF	0.8 [0.7–1]	0.6 [0.5–0.8]	0.6 [0.5–0.8]	0.6 [0.5–0.7]	0.6 [0.5–1.6]	0.46
HPF	1 [0.6–1.2]	0.7 [0.5–0.8]	0.6 [0.5–0.7]	0.6 [0.4–0.7]	0.7 [0.5–0.8]	
Phosphate (mg/dl)						
SF	2.7 [2–3.5]	2.9 [2.3–3.4]	3 [2.5–3.3]	3.5 [2.7–3.8]	3.5 [2.8–4.1]	0.41
HPF	3.1 [2.5–4]	2.9 [2.1–3.6]	3.5 [2.5–4]	3.4 [2.6–4.1]	3.6 [3.3–4]	
Proteins (g/L)						
SF	4.7 [3.9–6.1]	5.2 [4.7–5.6]	5.8 [5.2–6.3]	6.1 [5.3–6.5]	5.8 [5.2–6.4]	0.07
HPF	5.3 [5–5.7]	5.4 [5, 6]	5.8 [5.2–6.6]	6.4 [5.7–7.3]	7.3 [5.6–8.3]	
Albumin (g/L)						
SF	2.9 [2.4–3.4]*	2.6 [2.2–2.9]	2.6 [2.1–2.9]	2.8 [2.3–3]	2.5 [2.1–2.9]*	0.03
HPF	3.3 [3–3.6]*	2.7 [2.5–3.1]	2.7 [2.3–3.1]	3 [2.8–3.3]	3.1 [2.6–3.7]*	

Data expressed ad median [interquartile range]

The overall *p* value refers to the comparison between the whole time series of changes SF versus HPF

* *p* < 0.05 for the comparison of SF versus HPF at each time point

See abbreviations in the text

## Discussion

In our study, we adopted ultrasonography for the evaluation of quantitative and qualitative changes of skeletal muscles in a homogeneous group of young trauma patients who were previous healthy, well nourished and physically active. A previous similar study published few years ago [4] was focused on a heterogeneous group of critically ill patients with only 25% of them being trauma victims. All previous clinical studies with muscle ultrasonography have been conducted in mixed ICU populations that included medical and surgical, as well as acute and chronic, critically ill patients [4, 13, 19]. On the contrary, in our study there were no confounding factors such as old age, comorbidities, cancer and long-term use of medications.

### Quantitative changes of skeletal muscles

In ICU patients, the daily amount of muscle loss—as estimated by US—is reported to range between 6 [20] and 12.5% between day 1 and 7 [4]. Muscle wasting correlates with the ICU length of stay [16] and can be predictive of long-term functional disability [21]. Several factors contribute to muscle wasting over the course of the critical illness, both in the acute and chronic phase: inflammation, neuroendocrine stress response, immobilization, impaired microcirculation and denervation (in the acute phase); infections, nutritional deficiency, hyperglycemia, drugs [22] (in the late phase). Other predisposing factors are: age, baseline muscle function, nutritional status, comorbidities (COPD, renal and heart disease, cancer) [22, 23]. Finally, some data suggest that also parenteral nutrition may worsen muscle function [24].

A retrospective study demonstrated a correlation between hospital mortality and skeletal muscle mass, as estimated by abdominal CT scan [2]: this is particularly evident in trauma patients [25], but apparently not in ICU patients with acute lung injury [26].

Ultrasound has been used to rate the loss of skeletal muscles in patients with orthopedic trauma, COPD, cancer and neuromuscular disorders [13, 27, 28]; indeed, it appears as an emerging field of interest in ICU. Ultrasonography is more accurate than anthropometric measurements and has been shown to closely correlate with the data obtained by MRI and CT scan [19, 29], with the advantage of being less expensive, less time-consuming and safer, since it does not imply radiation exposure. Though some studies have shown a good intra-rater and inter-rater reliability for US measurement of muscle CSA or thickness in adult critically ill patients [4, 30], the matter is still somehow controversial [31, 32].

All of our trauma patients (100%) experienced severe muscle mass loss, as estimated by CSA. Almost half (45%) of RF muscle mass was lost by day 20 with the greatest reduction (21%) occurring after day 15. In a previous work [4], a 17.7% reduction in RF cross-sectional area was shown in a group of mixed ICU patients from day 1 to day 10, with the major loss occurring during the first 7 days. We found a less important reduction in AT cross-sectional area (22%) by day 20.

The exact underlying mechanisms of dissimilar magnitudes of losses in different muscle groups are still unknown. In both rodent and human models, the rate and magnitude of muscle loss seem to depend on both muscle type and degree of inactivity [33, 34]. In experimental and clinical models of lower limb immobilization, muscle loss is greater in the extensor muscles (soleus and gastrocnemius). This is consistent with the greater muscle loss we report in RF (extensor muscle) as compared to AT (flexor muscle). Also, RF is a power muscle made up predominantly of type II fast-twitch fibers, while AT muscle composition is mainly made of type I slow-twitch fibers [35]. The preferential loss of a certain kind of muscle fibers might be a crucial determinant of long-term outcome, especially in the development of ICU-acquired weakness and in success of physical rehabilitation. Muscle weakness is usually symmetric and predominates in the proximal part of the limbs (shoulders and ankles) [36]. Laboratory model of ischemic injury has shown that muscle with predominance of fast-twitch fibers had significantly greater necrosis than those richer in slow-twitch fibers [37]. Immobilization studies have shown a preferential loss of type II fibers and conversion of fiber typing from type I to type II in postural muscles [38].

### Qualitative changes of skeletal muscles

Several studies have demonstrated that pathological muscle changes (such as fatty infiltration, atrophy and intramuscular fibrosis) can be detected by ultrasound. Alteration of muscle echogenicity may be ascribed to muscle edema (in the early phase) but also to fibrosis and fatty degeneration (in the late phase). These latter findings may be an indicator of quantitative loss of muscular myofibers and disruption of muscle architecture and may correlate with impaired muscle function [18]. Since edema cannot alter the bone signal in contrast to fibrous tissue, changes in muscle echogenicity are related to the fibrous tissue content and with specific structural damage in muscle architecture as seen with muscle biopsies [4] or with muscle magnetic resonance imaging [18]. Structural muscle changes detected by the increased echogenicity have been correlated with measures of muscle strength and function [39].

In our study, echogenicity was quantified by the Heckmatt Scale (Table 5), previously used in the critically ill setting by Grimm [18]. A higher grade of echogenicity with reduced bone signal correlates with the severity of myopathy [17]. Of course, the use of a semiquantitative method such as the Heckmatt Scale may be biased by observer dependency and technical misinterpretation in contrast to objective, user-independent algorithms for image analysis such as computer-assisted quantitative grayscale analysis [40]. Though, the Heckmatt Scale has the relevant advantage of being a rapid and inexpensive bedside technique that can be easily used in the intensive care setting.

**Table 5:** Heckmatt Scale: visual grading scale to classify muscle echo intensity (Ref 17)

Grade	Ultrasound appearance
Grade 1	Normal
Grade 2	Increased muscle echo intensity with distinct bone echo
Grade 3	Marked increased muscle echo intensity with a reduced bone echo
Grade 4	Very strong muscle echo and complete loss of bone echo

Changes in muscle architecture have been documented in previous studies on ICU patients and are associated with increased length of stay in ICU [4, 16, 18, 27, 29]. Changes in muscle echogenicity [4, 18] suggest an alteration of myofibers content, secondary to edema from capillary leak or inflammation. These data are confirmed by muscle biopsies on day 1 and 7 after ICU admission, showing muscle necrosis and macrophage infiltrate [4].

In our study, we found a progressive increase in both RF and AT echogenicity from day 5 on. An alteration of echogenicity was already evident in AT muscle soon after admission and tended to increase in the following weeks. The early alteration of echogenicity in AT but not in RF may have many explanations; probably, post-traumatic edema was more pronounced in the muscles of the lateral/posterior part of the limb (AT) than in the anterior area (RF).

### Muscle wasting and nutrition

An optimal provision of energy and protein has been regarded as an important factor improving the patient’s chance of survival and satisfactory clinical outcome [41]. Provision of an optimal amount of protein has been shown to improve the rate of protein synthesis in tissues with rapid turnover, though it did not reduce the catabolic response to injury [42, 43]. In one recent multicenter study [44], provision of at least 80% of the prescribed protein (i.e., 1 g/kg/day) reduced mortality in a ICU population. In patients on parenteral nutrition, protein delivery may be more important than caloric support in terms of short-term outcome [11].

In our study, we found no difference in muscle mass loss or in muscle echogenicity between patients fed with standard (SF) versus high-protein formulas (HPF): though, no conclusion can be drawn in this regard, since the study was not designed or powered to verify such hypothesis. Nonetheless, this finding may be consistent with previous studies showing that depletion of lean body mass, particularly skeletal muscle, is not influenced by nutritional support [45–47] and with studies showing an inverse correlation between the amount of protein delivery and the cross-sectional area of RF [4].

Immobilization and inflammation—rather than inadequate nutritional support—might be major determinants of loss of muscle. Inactivity is a potent stimulus to muscle protein breakdown and activation of the ubiquitin–proteasome pathway of proteolysis [48]. Immobility of limbs is quite common in ICU patients and is related to bed rest and sedation. Acute and chronic activation of inflammatory pathway is another potent stimulus for proteolysis [49]. Though, in our study we did not measure any index of inflammatory activity and we could not verify such contention.

## Conclusions

In conclusion, we found that ultrasonography was an easy, effective and practical tool for the daily estimate of changes in skeletal muscles and we confirmed the feasibility of such methodology in trauma patients. Our data show that early loss of muscle mass is particularly relevant also in young trauma patients and that extensor muscles such as rectus femoris are much more affected than flexor muscles (anterior tibialis). Such quantitative muscle loss is associated with an increased echogenicity, possibly associated with progressively impaired muscle function.

## Abbreviations

AP diam: anterior–posterior diameter
APACHE: acute physiologic and chronic health evaluation
AT: anterior tibialis
BMI: body mass index
BUN: blood urea nitrogen
COPD: chronic obstructive pulmonary diseases
CSA: cross-sectional area
CT: computerized tomography
GCS: Glasgow Coma Scale
GCS-M: Glasgow Coma Scale (motor)
GOS: Glasgow Outcome Scale
HPF: high-protein feeding formula
ICU: intensive care unit
ISS: Injury Severity Score
LL diam: latero-lateral diameter
MDR: multiple drug resistant
RF: rectus femoris
SF: standard feeding formula
SOFA: sequential organ failure assessment
US: ultrasound
